# Book review: cancer and the heart

**DOI:** 10.1186/s40959-020-00089-1

**Published:** 2021-01-04

**Authors:** Umesh C. Sharma, Saraswati Pokharel

**Affiliations:** 1 Department of Medicine, Division of Cardiology, Jacob’s School of Medicine and Biomedical Sciences, Buffalo, NY USA; 2 Clinical & Translational Research Center, 875 Ellicott Street, 14203 Buffalo, NY USA; 3 Department of Pathology, Division of Thoracic Pathology and Oncology, Roswell Park Comprehensive Cancer Center, Buffalo, NY USA

**Keywords:** Cancer, Heart, Book Review

## Background

The 5-year relative survival rate for all cancers combined has increased substantially since the early 1960s, from 39–70%. Currently there are approximately 20 million cancer survivors. With increased cancer survivorship, there is progressive increase in the incidence of both reversible and irreversible cardiotoxicities mainly due to ionizing radiation, chemotherapy and both targeted and non-targeted immunotherapies. This book, authored by a multidisciplinary team of cardiologists, oncologists, cardiac imagers and research scientists makes a sincere effort to integrate a somewhat fragmented body of scientific literature into a comprehensive textbook [1].

The primary scope of this book is to provide an integrative understanding of the major principles and practice of cardio-oncology. First 6 chapters are dedicated to explaining the pharmacological basis of cardiotoxicity induced by most commonly used cancer therapeutic agents including anthracyclines, trastuzumab and emerging checkpoint inhibitors. A separate chapter is dedicated for describing the cardiovascular toxicities associated with the use of angiogenesis inhibitors. In addition, it summarizes the pathology, clinical manifestations and potential management strategies of radiation-induced heart disease. This section provides a most exhaustive review of cancer-specific irradiation approaches and their subsequent effects on myocardium, coronary vasculature, heart valves and pericardium. Advanced imaging approaches for the tissue characterization of radiation-induced myocardial pathology have been elegantly described. This book, however, stops short of delving into the toxic effects of novel CART-T cell therapies, which are still emerging and the full extent of their toxic potentials are still being recognized.

Unlike many other conventional textbooks, this book cuts a fine balance between the principles and practice of various pathologies associated with cancer therapy. For example, there is in-depth analysis of arterial and venous thromboembolic diseases, infective endocarditis, and infiltrative process that are relevant in patients with cancers and toxicities associated with those cancer therapies. Common and emerging technologies for the diagnosis of cancer therapy induced cardiotoxicities including cardiac ultrasonography, magnetic resonance imaging have been widely discussed. The gated cardiac CT, though briefly mentioned in Chap. 11, could be expanded somewhat in the future editions.

The other unique aspect of this book is to dissect the clinically relevant issues in multiple dimensions including age, sex and risk-status. In particular, there is a strong focus on highlighting the cardiac considerations for treating cancer in infants, children, and in pregnant women. The management aspects are broadly covered to include exercise physiology, pre-operative risk assessment and psychosocial considerations in treating cancer patients with heart disease. For the advanced cardiac pathology and recalcitrant heart failure, there is ample emphasis on principles of cardiovascular interventions and cardiac transplantation. Importantly, the cardiac emergencies among cancer patients also remains an important focus of this book, with a caveat that there are not enough data to formulate specific management protocols for the patients with checkpoint inhibitor induced myocarditis and the potentially deleterious effects of emerging CAR-T therapies.

We are currently practicing in the era of fast-paced innovation and discoveries that inherently comes with ever growing list of cancer therapy with cardiotoxic potential. We are more and more relying on advanced imaging and biomarkers as tools for diagnosing cardiovascular complication associated with cancer therapy but there is a lack of predictive and prognostic indices. Limitations to what is likely to become standard of care could have been considered.

## Conclusion

In conclusion, this book will serve as a major reference for cardiologists involved in general cardiovascular practice, cardio-oncology, or advanced cardiovascular imaging. This will also be of value for clinical oncologists and mid-level providers taking care of patients with various cancers. The major strength of this book is that it encompasses common cardiovascular morbidities including arrhythmias and heart failure in a most comprehensive manner. Despite a sound focus on the fundamental biology and pathophysiology of cardiotoxicity, this book also finds ways to weigh on emerging clinical trials, cardiac function testing and QT/QTc monitoring protocols in relevance to the most recent clinical algorithms utilized by medical practitioners.

The authors have managed to produce this monumental work, which is succinct yet comprehensive, practice-oriented material that has a very readable style of writing. That being said, the approach has remained mostly encyclopedic. More didactic, case-based or problem oriented approach could have made this book even more useful.

## Data Availability

NA.
